# The Challenge of Pneumatosis Intestinalis: A Contemporary Systematic Review

**DOI:** 10.3390/jpm14020167

**Published:** 2024-01-31

**Authors:** Gennaro Perrone, Mario Giuffrida, Valentina Donato, Gabriele Luciano Petracca, Giorgio Rossi, Giacomo Franzini, Sara Cecconi, Alfredo Annicchiarico, Elena Bonati, Fausto Catena

**Affiliations:** 1 Department of Emergency Surgery, Maggiore Hospital, 43126 Parma, Italygiorgiorossi@yahoo.it (G.R.);; 2 Department of General Surgery, Maggiore Hospital, 43126 Parma, Italy; 3 Department of General Surgery, AUSL Parma, 43125 Parma, Italy; 4 Department of Emergency and Trauma Surgey, Bufalini Trauma Center, 47023 Cesena, Italy; faustocatena@gmail.com

**Keywords:** pneumatosis cystoides intestinalis, surgery, emergency, portal vein gas, radiology

## Abstract

Purpose: Pneumatosis intestinalis is a radiological finding with incompletely understood pathogenesis. To date, there are no protocols to guide surgical intervention. Methods: A systematic review of literature, according to PRISMA criteria, was performed. Medline and PubMed were consulted to identify articles reporting on the items “emergency surgery, pneumatosis coli, and pneumatosis intestinalis” from January 2010 up to March 2022. This study has not been registered in relevant databases. Results: A total of 1673 patients were included. The average age was 67.1 ± 17.6 years. The etiology was unknown in 802 (47.9%) patients. Hemodynamic instability (246/1673–14.7% of the patients) was associated with bowel ischemia, necrosis, or perforation (*p* = 0.019). Conservative management was performed in 824 (49.2%) patients. Surgery was performed 619 (36.9%) times, especially in unstable patients with bowel ischemia signs, lactate levels greater than 2 mmol/L, and PVG (*p* = 0.0026). In 155 cases, surgery was performed without pathological findings. Conclusions: Many variables should be considered in the approach to patients with pneumatosis intestinalis. The challenge facing the surgeons is in truly identifying those who really would benefit and need surgical intervention. The watch and wait policy as a first step seems reasonable, reserving surgery only for patients who are unstable or with high suspicion of bowel ischemia, necrosis, or perforation.

## 1. Introduction

In 1783, Johann Georg Du Vernoy described, for the first time, pneumatosis intestinalis (PI), subsequently named Pneumatosis cystoides intestinalis by Mayer in 1825 [1]. PI is a physical or radiographic finding; it suggests the presence of gas in the bowel wall. PI is distributed throughout the digestive tract involving the subserous and/or submucosa of the small and large bowel. The typical location of pneumatosis intestinalis is the descending and sigmoid colon [2].

The true incidence of PI is unknown but the growing use of CT scans has contributed to the increased detection of this radiographic finding [3,4,5].

To date, there are no protocols to guide surgical intervention. Several factors are related to different management approaches to PI [6].

First, the clinical significance of PI can vary from benign findings to pathologic life-threatening bowel ischemia and necrosis [7,8].

Second, the pathogenesis of PI is poorly understood. PI can be the clinical manifestation of several diseases (IBD, especially Crohn’s disease, immune reactions or infections, bacterial abscesses, suppurative cholangitis, and other conditions that may require surgical treatment as bowel obstruction, pseudo-obstruction, malignancies, diverticulitis, and paralytic ileus).

PI has been also related to portal venous gas (PVG), especially in case of intestinal ischemia requiring emergency surgery.

PI and its wide range of clinical manifestations and etiologies represent a challenge for physicians, and especially for surgeons, the choice of the right treatment is not so easy.

The treatment depends on several factors. Suspected etiology and clinical and radiological presentation are the main factors behind the choice of treatment. Treatment can vary from simple drug discontinuation to open abdomen [2,3,4,5].

Literature reports about PI are typically case reports or small case series. Only a few cohort studies with a high number of included patients have been reported.

A systematic review of the literature of the last years was performed to evaluate the factors behind the choice of treatment and the real need for surgery in patients with pneumatosis intestinalis.

## 2. Methods

### Design

**Figure 1:**
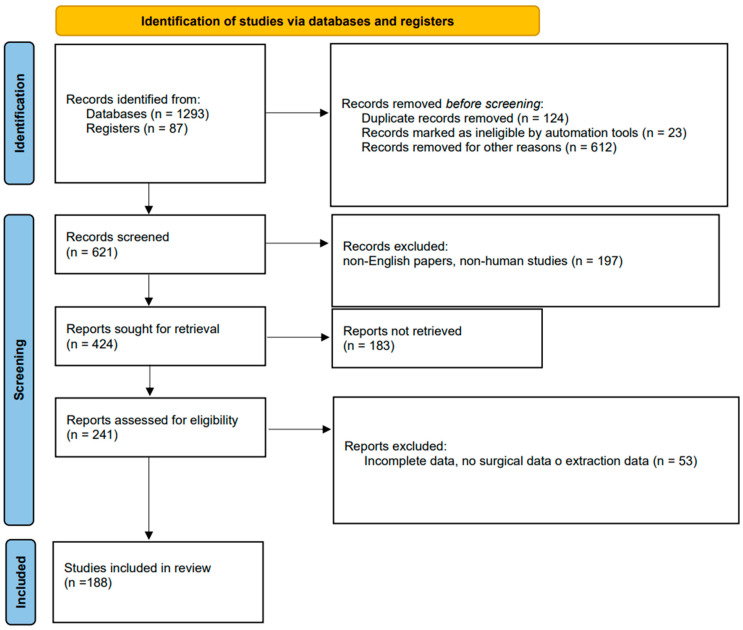
PRISMA flow-chart.

An extensive bibliographic search of the literature was performed according to modified PRISMA 2020 guidelines (Figure 1). The study was not registered.

All stages of study selection, data abstraction, and quality assessment were carried out independently by three reviewers (M.G. and A.A.). Any disagreements were resolved by consulting two other reviewers (F.C., G.P.).

Medline and PubMed were consulted in order to identify articles reporting the item “emergency surgery” from January 2010 up to March 2022 and then the Boolean operators “AND” and “OR” were used to mesh it with the following mesh terms: “pneumatosis coli”, “pneumatosis intestinalis”, “acute mesenteric ischemia”. Additional articles were searched by manual identification from the key articles.

We decided to include only papers from 2010 analyzing only a limited period of time. This choice was made to reduce the diagnostic and treatment biases of the past decades related to medical breakthroughs. We aim to take a picture of the etiology, diagnosis, and treatment of PI to understand why and when surgery must be performed or avoided.

Inclusion criteria: pneumatosis intestinalis of the small bowel and large bowel, articles in the English language. In the case of multiple papers from the same group of authors, an effort was made to identify duplicate papers. In the final dataset, every paper on pneumatosis intestinalis (cohort studies, retrospective and prospective studies) is included, also case reports and case series with complete data were included in the paper.

Exclusion criteria: Cases were excluded if the studies reported incomplete data or if the studies were not available in the English language or performed not in humans. Reviews were excluded.

Data relevant to the items of interest were abstracted. Several parameters were recorded and analyzed: gender, mean age, etiology, laboratory tests including cultural exams, symptoms, assessment of hemodynamic status (stable or unstable patients) diagnostic tests (colonoscopy, CT-Scan), location of PI, presence of pneumoperitoneum or portal vein gas (PVG) at diagnosis or delayed, treatment (conservative, surgical) and follow-up. Primary or secondary outcomes were analyzed.

Data analysis was performed using IBM SPSS Statistics 26.0. Univariate and multivariate analyses were performed.

Statistical analysis was obtained for the main descriptive indexes.

Quantitative data are expressed as mean or median ± standard deviation (SD). The qualitative data were elaborated as absolute frequencies, relative frequencies, cumulated frequencies, and percentages.

All factors were deemed to be statically significant at a *p*-value of less than 5% (*p* < 0.05).

## 3. Results

### 3.1. General Characteristics

After the assessment of abstracts and papers according to the inclusion criteria, 188 articles were included (Figure 1, Table 1).

A total of 1673 patients with pneumatosis intestinalis were included in the study, 773 (46.2%) were males and 581 (34.7%) were females. Gender was not reported in the remaining 319 (19.0%).

The average age was 67.1 ± 17.6 years. PI was related to bowel obstruction in 278 cases (16.6%), large bowel ischemia in 228 cases (13.6%), steroid therapy in 120 cases (7.1%), colonoscopy complications in 64 cases (3.8%), IBD complications in 54 patients (3.2%), monoclonal antibody drugs in 16 cases (0.9%). In 111 cases (6.6%), an underlying disease was found (chemotherapy complications, hyperganglionosis, trauma, sigmoid volvulus, necrotizing pancreatitis), and in 802 (47.9%) patients, the etiology was unknown.

Bacterial etiology was reported only in a few cases. Strongyloides stercoralis and Clostridium difficile were identified in 7 and 3 cases, respectively.

Demographic, pathological features, and etiology are detailed in Table 2 and Table 3.

The most common symptom was abdominal pain with distension in 396 patients (23.6%).

Hemodynamic instability was found in 246 (14.7%) patients.

### 3.2. Laboratory and Diagnostic Tests

Leukocytosis was observed in 585 patients (34.9%). CRP was documented in less than 207 (12.3%) patients and was elevated only in 61 (29.4%) patients. Elevated lactate level (≥2.0 mmol/L) was found in 359 patients (21.4%).

CT-scan of the abdomen was the most common diagnostic test in 1673 patients (100.0%). Plain X-ray was performed in 459 (27.4%) cases, colonoscopy was performed in 49 cases (2.3%).

PI of the small bowel was the most common site in 610 (36.4%) cases followed by colon and rectum in 497 (29.7%) cases.

The whole colon was involved in 107 (6.3%) patients. In 13 papers (566 patients, 33.8%), the exact location of PI was not reported.

Radiological findings of bowel ischemia (bowel wall thickening, mesenteric stranding, and ascites) were reported in 564 (33.7%) patients. Hepatic portal vein gas (PVG) was identified in 556 (33.2%) patients and pneumoperitoneum was radiologically reported in 301 (17.9%) cases.

### 3.3. Therapy

Conservative management was the most common treatment in 824 (49.2%) cases. Surgery was performed in 619 (36.9%) patients. Treatment was not reported in 230 (13.7%) cases.

Bowel rest, fluid administration, and antibiotics were the most common conservative treatments in 266 (15.8%) patients.

Every patient with PI related to IBD flare was successfully treated with a high dose of mesalamine and prednisone. PI caused by chemotherapeutic agents, monoclonal antibody drugs, and alpha-glucosidase inhibitors for diabetes were treated successfully with therapy discontinuation in 96 cases (11.7%).

Surgery was performed 619 (36.9%) times. Data about the surgical treatment of 227 (36.6%) patients were not reported.

Among the 619 who underwent surgery, bowel resection was the most common treatment in 237/619 (38.2%) cases.

Laparoscopic/laparotomy exploration without bowel resection was reported in 155 (25.0%) cases (Table 4).

Among the 866 (41.4%) patients with PI and confirmed etiology, 308 (35.5%) underwent surgery. Bowel resection was performed in 149 patients (48.3%) due to organic disease (volvulus, intussusception, Ogilvie’s syndrome, bowel obstruction, etc.). Bowel resection was not necessary in 54 (17.5%) patients. In 105 cases (34.0%), surgical treatment was not specified.

Among the 802 (58.6%) patients with unknown etiology, 311 (38.7%) underwent surgery, Bowel resection was performed in 88 patients (28.2%). Bowel resection was not necessary in 101 (32.4%). In 122 cases (39.2%), surgical treatment was not specified.

Among the 556 (33.2%) patients with PVG, 187 (33.6%) underwent surgery for bowel ischemia, necrosis, or perforation.

Death was reported in 390 (23.3%) cases, and 293 (75.1%) occurred in patients with critical conditions at hospital admission or during the first day after admission, where only supportive therapy was given. A total of 41 (10.5%) deaths were related to other causes. During the follow-up of the 155 cases treated with laparoscopic/laparotomy exploration alone without bowel resection were not reported as deaths.

Surgical management was significantly higher in unstable patients, with bowel ischemia signs, lactate levels greater than 2 mmol/L, and PVG (*p* = 0.0026).

Hemodynamic instability was reported in 246 patients (14.7%). Data about the clinical status of patients have not been reported in 309 (18.4%) patients.

Hemodynamically unstable patients were significantly associated with bowel ischemia, necrosis, or perforation (*p* = 0.019).

Higher mortality was significantly related to unstable patients, lactate levels greater than 2 mmol/L, and bowel ischemia signs (*p* = 0.031) but not with PVG (*p* > 0.05).

## 4. Discussion

Pneumatosis intestinalis is a radiological sign that shows several diagnostic and treatment issues.

Treatment can be a lifesaving decision and often the timing for surgical intervention is wrong. Clinical evolution of PI can often be unpredictable, it is responsible for a difficult treatment decision-making process that requires careful evaluation of every variable.

PI can be divided into primary PI (15% of all PI cases) and secondary PI representing 85% of cases. PI can be also divided into pathologic and asymptomatic PI [197].

Secondary PI has been attributed to endoscopic procedures, immunological disturbances, bowel mucosal disruptions, and intra-abdominal pathologies.

Pneumatosis intestinalis is a radiographic phenomenon produced by underlying diseases, which can vary widely. The pattern or extent of PI does not necessarily correlate with the severity of the symptoms or of the underlying disease. The same etiology can lead to both asymptomatic or pathologic PI, the PI severity depends on several factors, but there are no specific findings for pathological and asymptomatic PI [193,195,198,199].

The etiology, both for primary and secondary PI, remains unclear. More than 60 causative diseases and conditions have been identified, but the specific pathophysiology remains unknown [11,16,20,26,127,138,173].

Two pathogenetic hypotheses have been proposed, the mechanical and bacterial theories.

The mechanical theory hypothesizes that gas dissects into the bowel wall from the bowel lumen to some mechanism, causing increased overpressure, such as a bowel obstruction.

The bacterial theory proposes that gas-forming organisms produce gas within the bowel wall, entering the submucosa through mucosal rents or increasing mucosal permeability.

Different laboratory tests (CRP, LDH, and CPK) were reported to be elevated in the case of PI, especially in bowel ischemia, but their role in the diagnosis of pathologic PI is limited because they can be also elevated in systemic inflammatory reactions [47,152,162,192,200].

The patient’s personal history is mandatory in order to discover an underlying cause of PI, as suggested by our results where PI etiology was identified in 52% of the patients (recent endoscopy, diabetes therapy, steroid therapy, IBD, etc.).

Many studies have attempted to create algorithms for PI management. These algorithms may be difficult to apply clinically, especially when the patient requires immediate evaluation. Several studies have investigated the role of risk factors (hypotension, peritonitis, renal failure, serum lactate levels, older age) as predictors of a compromised bowel and the probable need for surgery [5,153,154,193,195,201].

The benign causes of PI usually result in mild or even no abdominal symptoms. In these patients, there are often no CT abnormalities other than the diagnosis of pneumatosis intestinalis.

CT findings can lead to an overtreatment of patients with PI. Portal venous gas has been traditionally associated with bowel necrosis, but our results do not suggest that PVG is always related to bowel ischemia. Among the 556 patients with PVG, 33.6% underwent surgery for bowel ischemia, necrosis, or perforation [9,10,11,12,13,14,15,16,17,18,19,20,21,22,23,24,25,26,27,28,29,30,31,32,33,34,35,36,37,38,39,40,41,42,43,44,45,46,47,48,49,50,51,52,53,54,55,56,57,58,59,60,61,62,63,64,65,66,67,68,69,70,71,72,73,74,75,76,77,78,79,80,81,82,83,84,85,86,87,88,89,90,91,92,93,94,95,96,97,98,99,100,101,102,103,104,105,106,107,108,109,110,111,112,113,114,115,116,117,118,119,120,121,122,123,124,125,126,127,128,129,130,131,132,133,134,135,136,137,138,139,140,141,142,143,144,145,146,147,148,149,150,151,152,153,154,155,156,157,158,159,160,161,162,163,164,165,166,167,168,169,170,171,172,173,174,175,176,177,178,179,180,181,182,183,184,185,186,187,188,189,190,191,192,193,194,195,196].

Peritoneal symptoms are usually reported in patients with life-threatening causes of PI.

Age ≥ 60 years, white blood cell count of >12, emesis, diarrhea, bloody stools, abdominal pain, constipation, weight loss, and tenesmus have been associated with life-threatening PI [92,202,203,204].

The treatment of pneumatosis intestinalis must focus on the underlying disease rather than on the radiographic sign itself. Surgery could be avoided when a non-organic etiology has been discovered. In this study, bowel resection was performed in 149 patients (48.3%) due to organic disease (volvulus, intussusception, Ogilvie’s syndrome, bowel obstruction, etc.). In 155/392 (39.5%) cases, surgery was performed without the identification of intraoperative pathological findings.

The treatment decision-making should be based on different points of view: the clinical status of patients, the presence of an underlying condition, the need for emergency surgery, and the possibility of simple observation and re-evaluation [103,104,105,106,107,108,109,110,111,112,113,114,115,116,117,118,119,120,121,122,123,124,125,126,127,128,129,130,131,132,133,134,135,136,137,138,139,140,141,142,143,144,145,146,147,148,149,150,151,152,153,154,155,156,157,158,159,160,161,162,163,164,165,166,167,168,169,170,171,172,173,174,175,176,177,178,179,180,181,182,183,184,185,186,187,188,189,190,191,192,193,194,195,196].

The timing and the decision process are crucial for the patient’s outcome.

The first step remains patient physical examination.

Unstable patients with signs of sepsis and symptoms of shock are most often associated with mesenteric ischemia, bowel necrosis, or bowel obstruction, as suggested by our findings. The outcome for these patients is most unfavorable among patients with PI. Surgical exploration has been performed in almost all cases of instability.

The second step is the identification of an underlying disease that may guide the treatment choice. An accurate anamnesis is fundamental to identifying and treating several diseases or conditions related to PI.

The third step includes the need for surgery. This is the sore point of PI treatment.

When an organic disease has been identified (bowel obstructions, intussusception, or volvulus) surgery remains the main treatment option, and also unstable patients could benefit from surgical exploration.

For stable patients without organic disease, a watchful waiting approach may be more indicated. The possibility of simple observation and re-evaluation should be considered, especially in stable patients with unknown etiology.

Instrumental findings of PVG and massive PI alone in stable patients are not mandatory for surgery. Another approach to stable patients could consist of initial laparoscopic exploration in patients with one or more signs of bowel ischemia or necrosis. Laparoscopy as the first step could avoid unnecessary laparotomy.

## 5. Conclusions

Our findings suggest and confirm the challenges associated with the appropriate treatment of patients with pneumatosis intestinalis. Many variables should be considered in the approach to patients with pneumatosis intestinalis. The treatment of patients with pneumatosis intestinalis is a lifesaving decision and the timing for surgical intervention is crucial. Accurate personal history of patients is fundamental for the management. Considering the wide range of causes and outcomes of pneumatosis intestinalis, the watch and wait policy as a first step could be reasonable in selected cases.

Surgery remains mandatory in unstable patients and when an organic disease has been identified. Surgical options should be explored, especially laparoscopic exploration in non-responders to conservative management with high suspicion of bowel ischemia and necrosis. It is important to recognize pneumatosis intestinalis as a clinical sign and not as a diagnosis.

## Figures and Tables

**Table 1:** Papers included in the literature systematic review.

References	No. of Patients	Gender	Age	Diagnostic Test	Etiology	Location	Treatment
Ling 2019 [9]	1	M	64	CT-Colonoscopy-US	Unknown	Sigmoid Colon	Conservative
Gao 2019 [10]	4	M (3) F (1)	61.2 (Mean)	CT (4)	IBD	Cecum (2) Ascending colon (2)	Conservative
Wang 2018 [11]	6	M (2) F (4)	55.5 (Mean)	CT (4)-Colonoscopy (4)-US (1)	Unknown (2)-glucocorticoid-TCE	Small bowel (1)-sigmoid (2)-descending colon (1)-transverse colon (1)-rectum	Conservative
Lee 2017 [12]	1	F	68	CT-Colonoscopy	Sunitinib	Small bowel-cecum	Conservative (Sunitinib suspension)
Wu 2013 [2]	1	M	70	CT-US	Unknown	All colon	Conservative
Amin 2020 [13]	1	M	61	CT-PET	Unknown	Descending colon	Conservative
Göbel 2019 [14]	1	M	46	Colonoscopy-X-ray	Unknown	Ascending Colon	Conservative
Smyth 2019 [15]	1	M	29	CT	Steroid therapy	All colon	Conservative
Lin 2019 [16]	1	M	65	CT-Colonoscopy	Acarbose	Sigmoid	Conservative (Acarbose suspension)
Cuevas 2019 [17]	1	F	65	CT	Unknown	Small bowel-ascending colon	Conservative
Kirmanidis 2018 [18]	1	F	82	CT-X-ray	Unknown	All colon	Conservative
Asahi 2018 [19]	1	M	67	CT	Sunitinib	Cecum	Conservative (Sunitinib suspension)
Vecchio 2018 [20]	1	M	86	CT	Myeloma Therapy	Transverse—descending colon	Conservative
Uruga 2018 [21]	1	F	71	CT-X-ray	Erlotinib	All colon	Conservative (Erlotinib suspension)
Akarsu 2018 [22]	1	M	65	CT-Colonoscopy	Unknown	Sigmoid	Conservative
Iwamuro 2018 [23]	1	F	74	CT-Colonoscopy	Pseudolipomatosis coli	Cecum-ascending colon	Conservative
Cho 2020 [24]	1	M	63	CT-Colonoscopy	Unknown	Sigmoid	Conservative
Tharmaradinam 2020 [25]	1	M	66	CT	Hyperganglionosis	Cecum-ascending colon	Right colectomy
Toyota 2020 [26]	1	M	59	CT	Decompression Sickness (DCS)	Transverse	Transverse resection-colostomy
Tsai 2019 [27]	1	M	46	CT-Colonoscopy	Meningititis	Ascending colon	Exploratory laparoscopy
Brighi 2019 [28]	1	M	70	CT	Unknown	All colon	Conservative
Tirumanisetty 2019 [29]	1	F	75	CT-X-ray	Unknown	Ascending colon	Right colectomy
Lee 2019 [30]	1	M	50	CT	Steroid therapy	-	Conservative
Arora 2014 [31]	1	M	68	CT-X-ray	Steroid therapy	-	Conservative
Abidali 2018 [32]	1	F	60	CT-Colonoscopy	AAA	Transverse colon	Endovascular AAA repair
Poor 2018 [33]	1	M	84	CT	Nintedanib	Cecum	Conservative (Nintedanib suspension)
Yamasaki 2019 [34]	1	F	63	CT-Colonoscopy	Unknown	All colon	Conservative
Peng 2019 [35]	1	F	60	Colonoscopy	Unknown	Descending colon	Conservative
Nukii 2019 [36]	1	F	69	CT	Osimertinib	Transverse colon	Conservative (Osimertinib suspension)
Chaundhry 2019 [37]	1	M	67	CT	Bevacizumab	Sigmoid	Conservative (Bevacizumab suspension)
González-Olivares 2019 [38]	2	F (2)	59 (Mean)	CT (2)	IBD (2)	Ascending (1) Ascending-transverse colon (1)	Conservative (2)
Kelly 2018 [39]	1	F	59	CT-X-ray	Unknown	All colon	Conservative
Shindo 2018 [40]	1	M	81	CT	Unknown	Small bowel-all colon	Conservative
Okuda 2018 [41]	1	F	91	CT	Sigmoid volvulus	Sigmoid	Sigmoid resection-colostomy
Liu 2017 [42]	1	M	55	CT-Colonoscopy	Intussusception terminal ileum	Ascending colon	Ileocecal resection
Zimmer 2018 [43]	1	M	45	CT-Colonoscopy	Unknown	Transverse colon	Conservative
Telegrafo 2017 [44]	1	M	54	CT	Steroid therapy	All colon	Conservative (Steroid suspension)
Liang 2018 [45]	1	F	66	Colonoscopy-Barium Enema	Unknown	Descending colon	Left colectomy
Ohkuma 2017 [46]	1	F	76	CT	Unknown	Descending colon	Conservative
Mikami 2017 [47]	1	M	72	CT-Colonoscopy	Salazosulfapyridine	All colon	Conservative (Salazosulfapyridine suspension)
Ribaldone 2017 [48]	1	F	59	CT-Colonoscopy-US	Unknown	Descending colon	Conservative
Sugihara 2017 [49]	1	F	48	CT	Unknown	Descending colon	Conservative
Robinson 2017 [50]	1	M	76	CT-X-ray	Ogilvie’s syndrome	Ascending colon	Right colectomy-ileostomy
Rachapalli 2017 [51]	1	M	50	CT	GVHD	Transverse colon	Conservative
Beetz 2019 [52]	1	M	60	CT-Colonoscopy	Steroid therapy	Transverse colon	Conservative (Steroid suspension)
Kanwal 2017 [53]	1	F	79	CT-X-ray	Colic perforation	Sigmoid	Left colectomy-colostomy
Suzuki 2017 [54]	3	M (1) F (2)	70.3 (Mean)	CT (3)	Voglibose (2) Unknown (1)	Small bowel (1)-all colon	Conservative (2 voglibose suspension)
Tsuji 2017 [55]	1	M	51	CT	Unknown	All colon	Conservative
Nishimura 2017 [56]	1	M	54	CT	Unknown	Ascending colon	Conservative
Faria 2016 [57]	1	M	69	CT	Chemotherapy	-	Conservative
Fujiya 2016 [58]	1	M	29	CT-Colonoscopy	Intussusception	Sigmoid	Intussusception reduction
Furihata 2016 [59]	1	M	81	CT-Colonoscopy	Unknown	Sigmoid	Conservative
Maeda 2016 [60]	1	F	80	CT-X-ray	Gefitinib	Small bowel-transverse colon	Conservative
Fraga 2016 [61]	1	F	66	CT-Colonoscopy	Unknown	Ascending colon	Conservative
Gassend 2016 [62]	1	M	72	CT-X-ray	Unknown	All colon	Subtotal colectomy
Castren 2016 [63]	1	F	74	CT	Unknown	Small bowel-all colon	Ileostomy
Waterland 2016 [64]	1	M	76	CT	GVHD	Ascending colon	Conservative
Keklik 2016 [65]	1	M	31	CT	Trauma	Small bowel-all colon	Conservative
Ksiadzyna 2016 [66]	1	M	64	CT-Colonoscopy	Acarbose	Ascending-transverse colon	Conservative (Acarbose suspension)
Vargas 2016 [67]	1	M	65	CT	5-FU	All colon	Conservative
Ling 2015 [68]	2	M (1) F (1)	60 (Mean)	CT (2)	Steroid therapy	Ascending colon (2)	Conservative (2)
Rottenstreich 2015 [69]	1	M	73	CT	Acarbose	Small bowel-ascending colon	Conservative (Acarbose suspension)
Balasuriya 2018 [70]	1	M	32	CT	Unknown	Ascending colon	Appendicectomy
Pülat 2015 [71]	1	M	33	CT-US-EGDS	Unknown	Descending colon	Conservative
Helo 2015 [72]	1	M	36	CT-X-ray	Unknown	All colon	Conservative
Castro-Poças 2015 [73]	1	M	65	Colonoscopy-US	Unknown	Sigmoid	Conservative
Ooi 2015 [74]	1	M	44	CT	Unknown	Descending colon	Hartmann’s procedure
Blair 2015 [75]	1	F	86	CT-X-ray	Unknown	All colon	Conservative
Chandola 2015 [76]	1	M	59	CT-X-ray	Unknown	Ascending-transverse colon	Conservative
Grimm 2015 [77]	1	M	21	CT	Unknown	Ascending colon	Conservative
Choi 2014 [78]	1	F	74	CT-X-ray	Unknown	Ascending-transverse colon	Conservative
Aziret 2014 [79]	1	M	62	CT-X-ray	Unknown	Small bowel-cecum	Ileocecal resection
Rodrigues-Pinto 2014 [80]	1	M	67	Colonoscopy	Unknown	-	Conservative
Jacob 2014 [81]	1	M	40	Colonoscopy	Unknown	-	LAR-ileostomy
Neesse 2015 [82]	1	M	81	CT-US	Unknown	Ascending colon	Right colectomy
Santos-Antunes 2014 [83]	1	M	73	Colonoscopy	Unknown	Ascending colon	Conservative
Martis 2014 [84]	1	F	77	CT	Unknown	Descending colon	Conservative
Krüger 2014 [85]	1	M	54	CT-US	Unknown	-	Conservative
Tseng 2014 [86]	1	F	50	CT-X-ray	Unknown	Ascending colon	Conservative
Rajpal 2014 [87]	1	F	56	CT-X-ray	Unknown	Ascending colon	Colic resection
Bamakhrama 2014 [88]	1	F	85	CT-colonoscopy-US	Unknown	Descending colon	Conservative
Chao 2014 [89]	1	F	40	CT	Unknown	Small bowel	Conservative
Jurado-Romàn 2014 [90]	1	M	87	CT	Unknown	Small bowel	Conservative
Pinto Pais 2014 [91]	1	F	43	CT-US	Unknown	Small bowel-cecum	Conservative
Lemos 2014 [92]	1	F	39	CT-colonoscopy-X-ray	Appendicitis	Cecum-ascending colon	Right colectomy
Qin 2014 [93]	1	M	29	CT-colonoscopy	Colonoscopy complication	Ascending-transverse colon	Conservative
Lim 2014 [94]	1	F	28	CT-X-ray	Unknown	-	Conservative
Nakajima 2013 [95]	1	M	52	CT-X-ray	Steroid therapy	-	Conservative
Bareggi 2014 [96]	1	F	32	CT	Unknown	-	Conservative
Fong 2014 [97]	1	M	85	CT-colonoscopy-X-ray	Sigmoid cancer	Ascending colon	Endoscopic stent
Zarbalian 2013 [98]	1	F	51	CT	Steroid therapy	-	Right colectomy
Lommen 2020 [99]	1	F	65	Colonoscopy-barium enema	Unknown	All colon	Conservative
Ezuka 2013 [100]	1	F	62	CT	Steroid therapy	Ascending colon	Conservative
Siddiqui 2013 [101]	1	M	35	CT-X-ray	Pancreatitis	Ascending colon	Conservative
Tanabe 2013 [102]	1	F	80	CT-X-ray	Alpha-Glucosidasy Inhibitor	-	Conservative (Alpha-Glucosidasy Inhibitor suspension)
Adar 2013 [103]	1	M	63	CT-colonoscopy- X-ray	Unknown	Descending colon	Alpha-Glucosidasy Inhibitor
Rahim 2013 [104]	1	M	39	CT	Steroid therapy	Cecum	Right colectomy
Liang 2013 [105]	1	M	88	CT	Unknown	Cecum-ascending colon	Conservative
Ponz de Leon 2013 [106]	1	M	54	Colonoscopy	Unknown	All colon	Total colectomy (FAP)
Mourra 2013 [107]	1	M	50	Colonoscopy	Unknown	All colon	Total colectomy (FAP)
Masuda 2013 [108]	1	F	68	Colonoscopy	Unknown	Ascending colon	Conservative
Kashima 2012 [109]	1	F	77	CT	Sorafenib	Unknown	None (death)
Aitken 2012 [110]	1	F	69	CT	Unknown	All colon	None (death)
Makni 2012 [111]	1	M	56	CT-X-ray	Unknown	Unknown	Conservative
Balbir-Gurman 2012 [112]	1	F	76	CT-X-ray	Unknown	Sigmoid	Conservative
Schieber 2012 [113]	1	F	19	CT-colonoscopy	IBD	Cecum-ascending colon	Conservative
Lee 2012 [114]	1	F	66	CT-X-ray	Gefitinib	All colon	Conservative (Gefitinib suspension)
Vijayakanthan 2012 [115]	2	M (2)	27.5 (Mean)	CT-X-ray	Imatinib	Cecum (1)-Transverse colon	Conservative (Imatinib suspension)
Chang 2012 [116]	1	M	85	CT-X-ray	Bowel Ischemia	Ascending colon	None (death)
Martin-Smith 2011 [117]	1	M	34	CT	Necrotizing pancreatitis	Cecum-ascending colon	Conservative
Hong 2012 [118]	1	F	75	CT	Unknown	Ascending colon	Laparoscopic exploration (ileostomy)
Hoot 2013 [119]	1	F	57	CT	Trauma	Ascending-transverse-sigmoid colon	Conservative
Shimada 2011 [120]	1	M	43	CT	Unknown	Cecum-ascending colon	Conservative
Iwasaku 2012 [121]	1	F	82	CT	Gefitinib	Ascending colon	Conservative (Gefitinib suspension)
Nancy 2013 [122]	1	M	22	CT	Colonoscopy complication	Ascending-transverse colon	Conservative
Sagara 2012 [123]	2	F (2)	48.5 (Mean)	CT (2)	Steroid therapy (1)	Sigmoid (1)	Colostomy (1)- Conservative (1)
Jarkowski 2011 [124]	1	M	73	CT	Sunitinib	Ascending-transverse colon	Conservative (Sunitinib suspension)
Wu 2011 [125]	1	F	67	CT-colonoscopy	Alpha-Glucosidasy Inhibitor	Ascending colon	Conservative (Alpha-Glucosidasy Inhibitor suspension)
Yoon 2011 [126]	3	M (1) F (2)	59.6 (Mean)	CT (3)	Cetuximab	Cecum (2)-ascending (2)-transverse colon (2)	Conservative (Cetuximab suspension)
Lioger 2012 [127]	1	M	67	CT	Collagen Disorders	Unknown	Unknown
Arenal 2011 [128]	1	F	18	CT	Unknown	Cecum	Conservative
Amrein 2011 [129]	2	M (1) F (1)	61.5 (Mean)	CT-colonoscopy	Unknown	Ascending colon (2)	Right colectomy (1) Conservative (1)
García-Castellanos 2011 [130]	1	F	32	CT-colonoscopy	Unknown	Descending-sigmoid colon-rectum	Left colectomy
Strote 2012 [131]	1	M	57	CT-US	Unknown	Ascending colon	Small Bowel Resection-Superior Mesentery Artery Thrombectomy
Kim 2011 [132]	1	F	40	CT-colonoscopy	Unknown	Sigmoid	Conservative
Shimojima 2011 [133]	1	M	48	CT-X-ray	Glimepiride Voglibose	Ascending colon	Conservative (Glimepiride Voglibose suspension)
Wright 2011 [134]	1	F	42	CT-X-ray	Unknown	Cecum	Conservative
Marinello 2010 [135]	1	M	20	CT	LES	Unknown	Conservative
Pasquier 2011 [136]	1	F	96	CT	Unknown	Unknown	Conservative
Bamba 2010 [137]	2	M (1) F (1)	43.5 (Mean)	CT-colonoscopy-X-ray (1)	Colonoscopy complication	Cecum (1)-ascending (1)-transverse colon (1)	Conservative
Huang 2010 [138]	1	M	30	CT-X-ray	Transplantation complication	Ascending colon	Conservative
Liao 2010 [139]	1	M	48	CT-US	Colonic trauma	Ascending colon	Right colectomy
Chaput 2010 [140]	1	M	57	Colonoscopy-X-ray-Manometry	Unknown	Rectum	Conservative
Ong 2010 [141]	1	M	69	CT-X-ray	Volvulus	All colon	Total colectomy
Newman 2010 [142]	1	M	26	CT-X-ray	GVHD	Unknown	Conservative
Syed 2020 [143]	1	M	60	CT-colonoscopy	Clindamycin	Sigmoid	Conservative
Meini 2020 [144]	1	M	44	CT	COVID-19	Ascending colon	Conservative
Miwa 2020 [145]	1	M	58	CT-colonoscopy	Unknown	Ascending-transverse colon	Conservative
Kielty 2020 [146]	1	M	47	CT	COVID-19	Small bowel-cecum	Conservative
Lakshmanan 2020 [147]	1	M	72	CT	COVID-19	Ascending-sigmoid colon	Conservative
Hokama 2020 [148]	1	M	91	CT	Strongyloides Stercoralis	Small bowel-all colon	Conservative
Wang 2020 [149]	2	M (2)	90 (Mean)	CT	Unknown	Small bowel-all colon	Conservative
Ribolla 2020 [150]	1	F	65	CT	Unknown	Ascending colon	Laparotomy exploration
Zhang 2012 [151]	1	M	60	CT	IBD	Transverse-descending-sigmoid colon	Conservative
Ferrada 2017 [152]	127	-	57 (Mean)	CT (117)-X-ray (8)	Unknown	Small bowel (61)-cecum (40)-ascending (60)-transverse (17)-descending colon (12)-sigmoid (11)-rectum (3)	Surgery (70) Conservative (57)
Matsumoto 2016 [153]	70	M (38) F (32)	72 (Mean)	CT (70)	Unknown	Small bowel (42)-ascending (20)-descending colon (8)	Surgery (39) Conservative
Bani 2013 [154]	209	-	56.8 (Mean)	CT (209)	Obstruction (53)- ischemia (53)	Unknown	Surgery
DuBose 2013 [6]	500	M (283) F (217)	56.6 (Mean)	CT (500)	IBD (18)- Colonoscopy complication (57)	Small bowel (305)-colon (285)-rectum (3)	Surgery (199) Conservative (301)
Gupta 2020 [155]	1	F	81	CT	Unknown	Small bowel	Conservative
Muhammad Nawawi 2020 [156]	1	M	38	CT	Unknown	Small bowel	Conservative
Gomes 2020 [157]	1	F	90	CT	Sigmoid cancer	Small bowel	Surgery
Takimoto 2020 [158]	1	F	75	CT	M. avium-amyloidosis	Colon	Conservative
Lim 2020 [159]	1	M	68	CT	Unknown	Small bowel	Surgery
Fairley 2020 [160]	1	M	71	CT	Colonoscopy complication	Colon	Conservative
Molina 2020 [161]	1	F	72	CT-X-ray	Unknown	Small bowel	Surgery
Arai 2020 [162]	25	M (17) F (8)	75 (Mean)	CT	Unknown	Colon-Small bowel	Surgery (17) Conservative (8)
Police 2020 [163]	1	M	72	CT	Sigmoid volvulus	Sigmoid colon	Surgery (Sigmoidectomy)
Wheatley 2020 [164]	1	F	52	CT	Unknown	Small bowel	Surgery
Tsang 2019 [165]	2	M (1) F (1)	67.5 (Mean)	CT (2)	Unknown	Small bowel-ascending colon	Surgery (1) Conservative (1)
Chen 2019 [166]	1	M	63	CT-US	Unknown	Small bowel	Surgery
Kim 2019 [167]	2	M (2)	75.5 (Mean)	CT	Cardiac surgery	Small bowel	Suergery (2)
Furutani 2019 [168]	1	M	69	CT	Colic resection	Ascending colon	Conservative
Varelas 2019 [169]	11	M (9) F (2)	61 (Mean)	CT	Lactulose (9) Unknown (2)	Small bowel (1)-colon (11)	Surgery (2) Conservative (9)
Arai 2019 [170]	1	M	51	CT	Unknown	Small bowel	Surgery
Belkhir 2019 [171]	1	M	28	CT-US	Unknown	Small bowel	Surgery
Khan 2019 [172]	1	F	70	CT	Capecitabine	Small bowel	Surgery
Di Pietropaolo 2019 [173]	1	F	78	CT	Chemotherapy	Small bowel-ascending colon	Surgery
Perez Rivera 2019 [174]	1	M	19	CT	Previous gastrostomy	Small bowel-colon	Conservative
Ibrahim 2019 [175]	1	M	69	CT	Unknown	Small bowel	Surgery
Harris 2019 [176]	1	F	57	CT-X-ray	Jejunal lymphangioma	Small bower	Surgery
Bansal 2019 [177]	1	M	52	CT	Unknown	Small bowel	Surgery
Dhadlie 2018 [178]	2	M (2)	80.5 (Mean)	CT (1)-X-ray (1)	Unknown	Small bowel	Surgery (1) Conservative (1)
Sanford 2018 [179]	4	M (2) F (2)	77 (Mean)	CT (4)	Unknown	Small bowel (2)-cecum (2)-ascending-sigmoid colon	Surgery (3) Conservative (1)
Guan 2018 [180]	1	F	78	CT	Systemic sclerosis	Small bowel-colon	Surgery
Gray 2018 [181]	1	F	64	CT	Unknown	Small bowel	Surgery
Fujimi 2016 [182]	1	M	55	CT	Nilotinib	Small bowel	Conservative
Yamamamoto 2020 [183]	1	F	70	CT-colonoscopy	Unknown	Ascending colon	Conservative
Dibra 2020 [184]	1	F	60	CT-X-ray	Unknown	Small bowel	Surgery
Fukunaga 2022 [185]	1	F	81	CT	Cardiac surgery	Small bowel	Surgery
Furtado 2022 [186]	1	F	81	CT	Unknown	Small Bowel	Conservative
Sharp 2022 [187]	1	M	61	CT-X-ray	Pseudomonas aeruginosa	Small bowel	Conservative
Gefen 2022 [188]	1	M	40	CT-X-ray	Steroid therapy	Ascending colon	Surgery
Yadzi 2021 [189]	1	M	30	CT	Ileal volvulus	Small bowel	Surgery
Yeo 2021 [190]	2	M	71.5 (Mean)	CT-X-ray	Steroid therapy Chemotherapy	Small bowel	Surgery (1) Conservative (1)
Brocchi 2021 [191]	8	M (5) F (3)	65.5 (Mean)	CT	Chemotherapy	Small bowel (2) Colon (6)	Surgery Conservative
Yamamoto 2021 [192]	1	M	43	CT	Steroid therapy	Colon	Conservative
Della seta 2021 [193]	290	M (171) F (119)	66.7 (Mean)	CT	Obstruction (110) Ischemia (94) Volvulus-Intussusception (43) Sepsis (78)	Unknown	Surgery (155) Conservative (135)
Adachi 2020 [194]	21	M (12) F (9)	80.1 (Mean)	CT	Steroid therapy (3) Chemotherapy (1) Alpha-Glucosidasy Inhibitor (1) Unknown (16)	Small bowel (12) Colon (6)	Conservative
Epin 2022 [195]	58	M (37) F (21)	72.0 (Mean)	CT	Unknown	Small Bowel	Surgery (25) Conservative (33)
Treyaud 2017 [196]	149	M (96) F (53)	64.0 (Mean)	CT	Obstruction (10) Ischemia (80)	Small Bowel (72) Colon (96)	Surgery (51) Conservative (98)

**Table 2:** Demographic and pathological features of the studied population.

Parameters	Analyzed Variable	No, %	Mean ± SD
Sex	Female	581, 34.7%	
	Male	773, 46.2%	
	Not reported	319, 19.0%	
Mean age (years)	All considered patients		67.1 ± 17.6
Etiology	Known	871, 52.0%	
	Unknown	802, 47.9%	
Diagnostic findings	Signs of bowel ischemia	564, 33.7%	
	Portal vein gas	556, 33.2%	
	Pneumoperitoneum	301, 17,9%	

SD: Standard deviation.

**Table 3:** Etiology of Pneumatosis Intestinalis.

Etiology	(No. of Patients, % *)
Bowel Obstruction	278, 16.6%
Steroid Therapy	120, 7.1%
Colonoscopy Complications	64, 3.8%
Large Bowel Ischemia	228, 13.6%
IBD complications	54, 3.2%
Monoclonal Antibody Drugs	16, 0.9%
Other	68, 4.0%
Unknown	802, 47.9%
Total PI (n.)	1673, 100%

PI: Pneumatosis Intestinalis. * Percentage refers to the total of patients for respective etiology.

**Table 4:** Therapy.

Therapeutic Approach	No (1673), 100% *
NOM	824, 49.2%
Drugs discontinuation	96, 11.7%
Antibiotics-TPN	166, 20.1%
IBD therapy	54, 6.5%
NR NOM	508, 61.6%
Surgery	619, 36.9%
Bowel resection	237, 38.2%
Laparoscopic/Laparotomy exploration (no resections)	155, 25.0%
NR Surgical treatment	227, 36.6%
NR	230, 13.7%

NOM: Non-operative management. NR: Not reported. * The % refers to the total patients of NOM and Surgery procedures.

## Data Availability

The data that support the findings of this study are available from the corresponding author, upon reasonable request.

## Abbreviations

PI: Pneumatosis intestinalis; PVG: Portal vein gas.
